# Topographical Central Island-Like Pattern After 24 Hrs of Continuous Intraocular Pressure Monitoring with a Contact Lens Sensor

**DOI:** 10.2147/IMCRJ.S232659

**Published:** 2020-02-10

**Authors:** Hiroshi Toshida

**Affiliations:** 1 Department of Ophthalmology, Juntendo University Shizuoka Hospital, Izunokuni, Shizuoka, Japan

**Keywords:** contact lens sensor, CLS, triggerﬁsh, topography, orthokeratological effects, central island

## Abstract

With development of the contact lens sensor (CLS), it has become possible to monitor the intraocular pressure (IOP) for 24 hrs continuously. Wearing of CLS often brings blurred vision with transient aggravation of myopia and changes in corneal shape. The author, a 51-year-old man with myopic astigmatism, wore a CLS for 24 hrs on the right eye, and the fellow eye served as a contra-lateral control eye. After wearing, his corrected visual acuity on the right eye decreased from 20/16 to 20/25 with blurred vision, and subjective spherical power and cylindrical power aggravated. Topographical analysis revealed that the instantaneous power increased on the central cornea but decreased on the mid-peripheral cornea. Differential instantaneous map of pre- and post-wearing CLS showed a specific pattern similar to the central island pattern, which is known as the results of steeper fitting of the orthokeratology lens. A surface imprint was observed on the bulbar conjunctiva, corresponding to the edge of the contact lens. These findings seemed due to orthokeratological effects by the steeper fitting of CLS. All of them resolved within 24 hrs after the removal of the CLS.

## Introduction

It is well known that the intraocular pressure (IOP) varies throughout the day not only glaucoma patients but normal subjects.^1^,^2^ In patients with glaucoma, the IOP is usually measured once at a clinic, so the pattern of IOP values during daily life is unknown. Diurnal variation of the IOP is observed in people with healthy eyes as well as patients with glaucoma, and the pattern of such diurnal variation differs widely between individuals and according to body posture.^1^–^4^ To assess the diurnal variation of the IOP, it used to be necessary for a patient to be admitted to a hospital and undergo repeated IOP measurement over 24 hrs. This method of evaluation placed a heavy burden on both patients and ophthalmologists.

After the SENSIMED Triggerfish^®^ contact lens sensor (CLS [Sensimed AG, Lausanne, Switzerland]) was developed in 2009, it became possible to measure the IOP continuously over 24 hrs by wearing a CLS.^5^,^6^ Therefore, availability of the CLS has reduced the burden of investigating the IOP for both patients and ophthalmologists. The CLS is placed on the eye to be tested and monitors the changes of corneal curvature induced by changes of the IOP, while transmitting measurements to a “Triggerfish^®^” receiver that records the data.^7^ This system can be used to detect the peaks of IOP.

Most of the previous studies were performed in participants with spherical power between −5 and 3 diopters (D) and cylindrical power 2D or less,^8^–^13^ awareness of blurred vision is one of the main frequent adverse events with CLS.^8^,^10^,^14^–^16^ Although the CLS is available in three curvature radius sizes: steep, medium, and flat,^8^ curvature radius size of CLS is selected based on the measured corneal curvature radius of the patient’s eyes. As the fitting of CLS generally tight, wearing of a CLS often brings transient aggravation of myopia with changes in corneal shape and refractive power.

In the present study, a participant who had myopic astigmatism showed a specific pattern similar to the central island pattern^17^ caused by the steeper fitting of orthokeratology lens in the differential instantaneous map of pre- and post-wearing lenses. This topographical pattern showing a perfectly centered area of central corneal steepening, surrounded by a “moat” of marked flattening seemed due to orthokeratological effects by tight fitting of CLS.^8^

## Materials and Methods

The author, a 51-year-old healthy man who had myopic astigmatism without glaucoma (author) participated in the present study. On the day before wearing the CLS, after checking the eyes, the objective refraction value measured by automated refraction and keratometry (ARK-1a, Nidek, Gamagori, Japan), subjective refraction value and visual acuity were examined. The IOP was measured by noncontact tonometer (Icare^®^ PRO tonometer, Icare Finland Oy, Vantaa, Finland), and the shape of the cornea and corneal thickness were determined by anterior-segment optical coherence tomography (AS-OCT, SS-1000 CASIA, Tomey, Nagoya, Japan) as the previous study.^17^ The average power and corneal thickness in paracentral, mid-peripheral and peripheral cornea shown on the AC-OCT map were also calculated by each 8 measurement points on the concentric circles with diameter of 3, 6 and 9mm, respectively.

A CLS was placed on the right eye of the subject at 5 p.m. to continuously measure the IOP for 24 hrs. In addition, the IOP of the left eye was measured every 3 hrs from 5 p.m. by noncontact tonometer. After 24 hrs (ie, at 5 p.m. on the next day), the CLS was removed from the right eye, and the same examinations performed before wearing the CLS were repeated. In addition, fluorescein staining and the rose Bengal staining of the cornea and conjunctiva were performed. All of these examinations were done again at 48 hrs after the start of CLS use.

Wilcoxon *t*-test was used to compare the results of average power and corneal thickness measured by AS-OCT before and after CLS wearing and significance was set at p<0.05.

## Results

**Figure 1:**
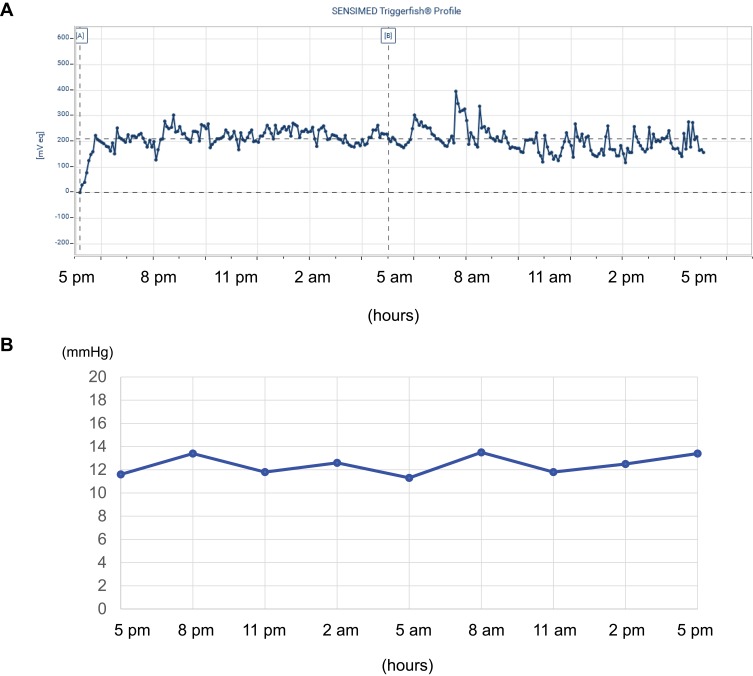
Results of IOP. **Notes:** (**A**) IOP measured by 24 hr continuous monitoring by the CLS in the right eye. (**B**) IOP measured by a noncontact tonometer every 3 hrs in the left eye. The IOP did not change markedly in both eyes during 24 hr period.

As the mean keratometry was 7.79mm, a CLS with a medium curvature of 8.7 mm was chosen by the manufacture’s manual. The results of IOP obtained by 24 hr continuous monitoring using the CLS in the right eye in Figure 1A and IOP by a noncontact tonometer every 3 hrs in the left eye are shown in Figure 1B. Before wearing the CLS, the IOP measured by a noncontact tonometer was 13.0 mmHg for the right eye and 11.6 mmHg for the left eye (Table 1). During the 24 hr period, the IOP did not change markedly in either eye, and the pattern of IOP variation corresponded to the “no significant acrophase” pattern described in past reports.^9^ Immediately after removal of the CLS from the right eye, the IOP measured by a noncontact tonometer was 13.6 mmHg for the right eye and 13.4 mmHg for the left eye.

**Table 1:** Results of Ophthalmological Examinations Before and After Wearing CLS

	Before	24 Hr After	p value
Intraocular Pressure (mmHg)			
Experimental side (right eye)	13.0	13.6	
Contra-lateral control side (left eye)	11.6	13.4	
Uncorrected visual acuity	2/20	2/20	
Corrected visual acuity	24/20	16/20	
Keratometry			
KM (D)	43.25	38.25	
Kmax (D)	45.00	38.75	
Kmin (D)	41.75	37.50	
cyl (D)	−3.25	−2.75	
Axis	178º	11º	
Subjective Refraction			
Spherical power (D)	−1.75	−3.75	
Cylinder power (D)	−1.75	−2.00	
Axis	180º	180º	
Spherical equivalent refractive error (D)	−2.63	−4.75	
Instantaneous Power (keratometric)			
Corneal center (D)	42.00	45.00	
Paracentral cornea (D)	42.55 ± 1.34	40.53 ± 4.46	
Midperipheral cornea (D)	41.50 ± 1.97	33.94 ± 4.04	p=0.0117*
Peripheral cornea (D)	36.35 ± 3.20	40.10 ± 7.18	
Corneal Thickness			
Corneal center (µm)	515	528	
Paracentral cornea (µm)	528.6 ± 15.1	533.8 ± 10.4	
Midperipheral cornea (µm)	554.8 ± 25.3	559.5 ± 32.8	
Peripheral cornea (µm)	591.8 ± 35.4	620.8 ± 48.5	p=0.0117*

**Notes:** Results are partially expressed as the mean, SD. *Wilcoxon *t*-test. **Abbreviations:** MK, mean keratometry; D, diopters; Kmax, maximum keratometry; Kmin, minimum keratometry.

**Figure 2:**
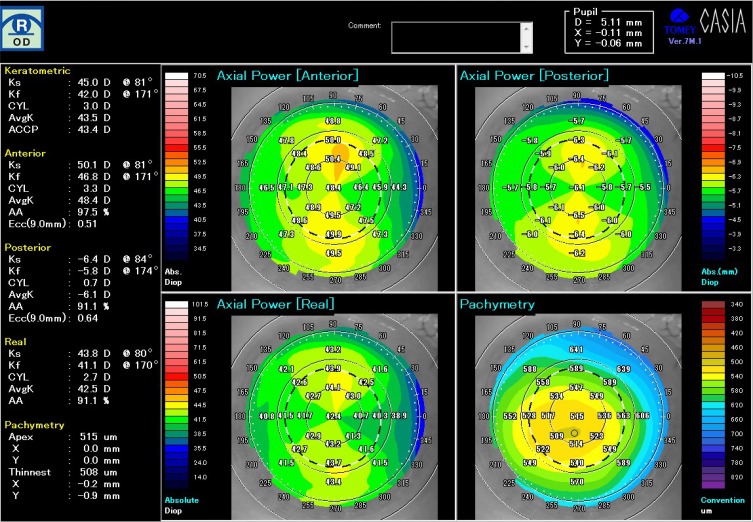
The topographic map of the axial power and pachymetry before placement of CLS.

**Figure 3:**
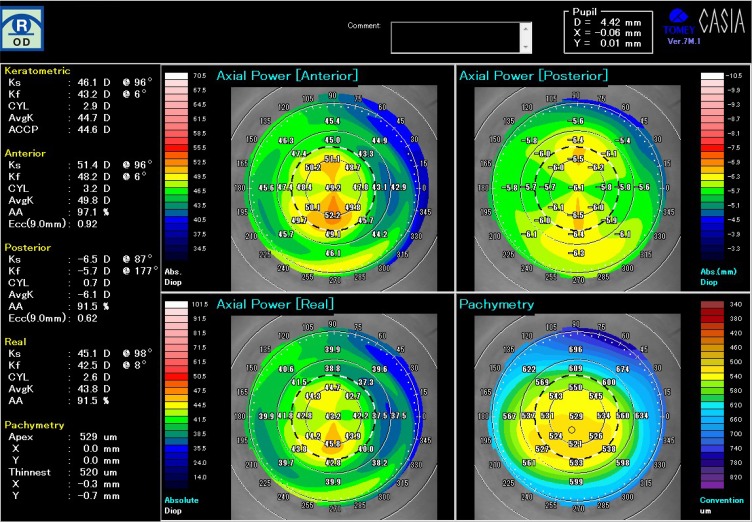
The topographic map of the axial power and pachymetry just after removal of CLS after 24 hr continuous monitoring of IOP.

**Figure 4:**
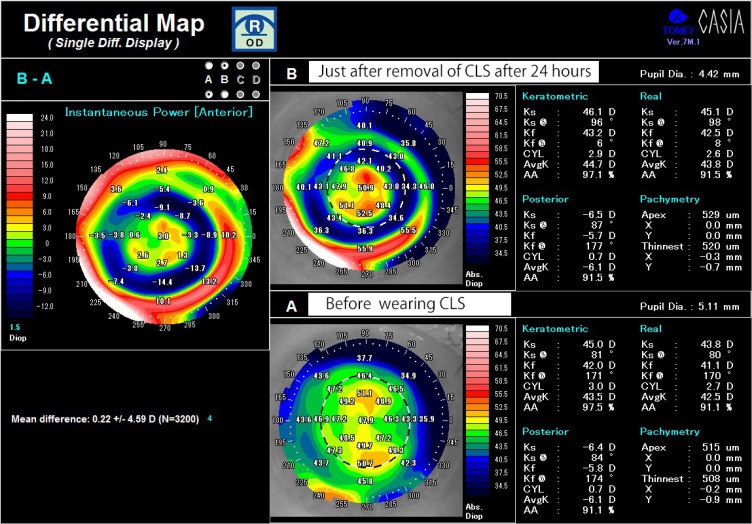
The differential map of the instantaneous power measured at pre-and post-wearing CLS. **Note:** It showed a specific pattern shaped to resemble central island pattern caused by steeper orthokeratology lens fitting.

His corrected visual acuity on the right eye decreased from 24/20 to 16/20 after wearing a CLS. With regard to subjective refraction, the spherical power was increased from −1.75D to −3.75D, and cylindrical power slightly increased from −1.75D to −2.00D after wearing the CLS for 24 hrs (Table 1). The spherical equivalent refractive error (D) was also increased from −2.63D to −4.75D. Figure 2 shows the data from the topographic map of the axial power and pachymetry before wearing CLS and Figure 3 just after removal of CLS at 24 hrs. There were obvious changes in the anterior and the real power. Instantaneous power also increased on the corneal center and decreased on the mid-peripheral cornea after wearing the CLS compared with baseline (Figure 4). The differential map (Figure 4) of the instantaneous power measured at pre-and post-wearing CLS showed a specific pattern similar to the central island pattern caused by steeper orthokeratology lens fitting. Thus, the corneal curvature became steeper and the cylindrical power was increased, so astigmatism was aggravated. While wearing the CLS and after its removal, the subject noted blurring of his vision. The central corneal thickness increased from 515 µm before wearing the CLS to 528 µm after 24 hrs, and corneal thickness averaged by 8 points in the peripheral cornea also increased from 591.8 ± 35.4 µm to 620.8 ± 48.5 µm statistically (p=0.0117, wilcoxson *t*-test). The power on the mid-peripheral cornea averaged by 8 points decreased statistically (p=0.0117, wilcoxson *t*-test). At 24 hrs after removing the CLS (ie, 48 hrs after the start of this experiment), most of the measured values returned to baseline as following; the visual acuity returned to 24/20, the spherical power to −1.75D, spherical equivalent refractive error (D) to −2.75D. Instantaneous power of keratometric in the mid-peripheral cornea to 41.59 ± 2.58 D and corneal thickness in the peripheral cornea to 598.8 ± 32.4µm.

**Figure 5:**
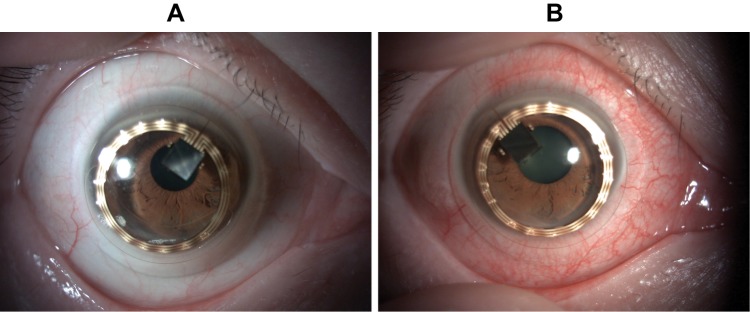
Ocular findings of the anterior segment at just placed the CLS and at 1 hr after starting. **Notes:** (**A**) CLS on the right eye just after placed. There was no hyperemia. (**B**) The CLS is fixed with the bulbar conjunctiva and mild bulbar conjunctival hyperemia was shown.

**Figure 6:**
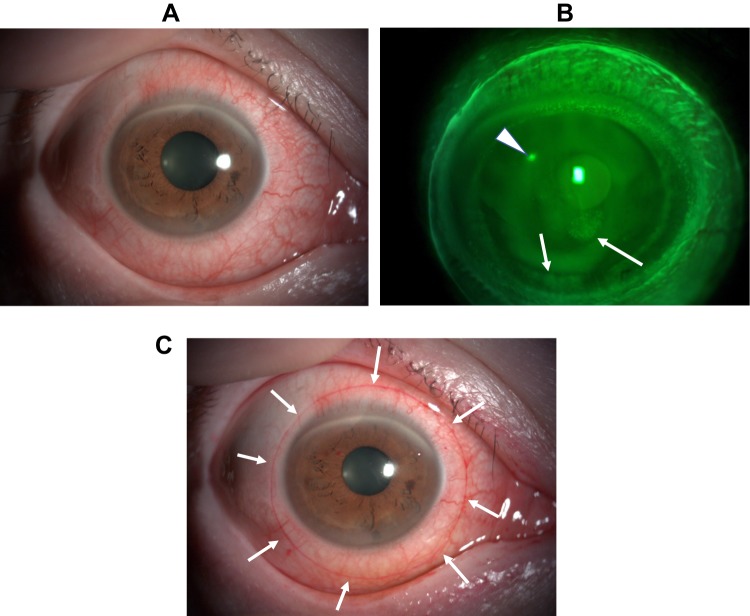
Ocular findings just after removal of the CLS following 24 hr continuous measurement of the IOP. **Notes:** (**A**) The bulbar conjunctiva showed obvious hyperemia. (**B**) Fluorescein staining. The superficial punctate keratitis was shown in superior temporal mid-peripheral (arrowhead) and inferior segment (arrow). (**C**) Rose Bengal staining. The surface imprint on the bulbar conjunctiva, corresponding to the edge of the CLS was observed (arrow).

The ocular findings at just starting CLS wear (A) and at 1 hr after CLS wear (B) is shown in Figure 5. The CLS is fixed with slight inferior displacement and mild bulbar conjunctival hyperemia can be observed. The ocular findings immediately after removal of the CLS following 24 hr continuous measurement of the IOP are shown in Figure 6A. The bulbar conjunctiva showed obvious hyperemia. The superficial punctate keratitis stained by fluorescein was positive in the superior temporal mid-peripheral segment of the cornea (arrowhead) and inferior segment (arrow) (Figure 6B). Rose Bengal staining revealed a surface imprint on the bulbar conjunctiva, corresponding to the edge of the CLS (Figure 6C). Both while wearing the CLS and after removal of the CLS, the subject noted intermittent itching of the eye. After removal of the CLS, the subject instilled 0.1% hyaluronic acid eye drops 3 times during the first 24 hrs. Both the objective findings and the subjective symptoms resolved within 24 hrs after removal of the contact lens, ie, within 48 hrs after the start of this experiment.

## Discussion

The first report on the 24 hr continuous measurement of the IOP using the CLS was published in 2011.^6^ Since then, measurements of the IOP obtained using the CLS in patients with glaucoma, healthy subjects, and patients with sleep apnea syndrome have been reported.^7^–^16^,^18^ However, there have been relatively a few reports about the influences of the CLS on the cornea.^13^,^19^–^21^ After wearing the CLS, the most common corneal/conjunctival finding is reported to be bulbar conjunctival hyperemia, followed by superficial punctate keratopathy and corneal epithelial erosion.^6^,^8^,^10^,^14^–^16^,^22^ In contrast, there have been few reports about the proper fitting of the CLS. In the present study, bulbar conjunctival hyperemia and superficial punctate keratopathy were noted after wearing the CLS, but these changes resolved within 24 hrs. It was thought that these changes occurred because the curvature of the CLS was too steep for the subject’s eye. In fact, the CLS stuck to the eye, so, the instillation of an anesthetic eye drop was required when the CLS was removed and forceps were also needed. After wearing the CLS for 24 hrs, visual acuity decreased with blurred vision, and aggravation of myopia and astigmatism was noted transiently, indicating that the corneal curvature had become steeper on the corneal center. It seemed to be due to steeper fitting of the CLS, and transient myopization is in agreement with the previous reports.^8^,^10^,^13^,^19^,^21^ Furthermore, topographic analysis in the present study revealed that the instantaneous power increased on the central cornea but decreased on the mid-peripheral cornea. Differential map of the instantaneous power comparing pre- and post-wearing of CLS showed a specific pattern was similar to the central island pattern caused by the steeper fitting of orthokeratology lenses.^8^,^17^ So, it is thought that both are caused by an orthokeratological effect with steeper lens fitting.

According to the manufacturer of the CLS, even if the curvature of the lens selected by measurement of the patient’s eye is too steep, the selected lens cannot be changed for another CLS with a different curvature. There are three curvature radius sizes for the CLS, which are termed steep, medium, and flat. Unlike the method for fitting a conventional soft contact lens (SCL), a CLS with a steep, medium, or flat curvature is selected by measurement of the patient’s corneal curvature radius. The manufacturer’s manual for the CLS specifies that a lens with a steep curvature of 8.4 mm, a lens with a medium curvature of 8.7 mm, and a lens with a flat curvature of 9.0 mm should be selected for eyes with a corneal curvature radius of 7.53 mm or less, 7.54–8.44 mm, and 8.45 mm or more, respectively. With regard to ordinary SCLs, if the fit of the selected lens is too tight, the prescription is generally changed for a new SCL with a larger curvature. With the Triggerfish system, however, there is no opportunity for correction of the CLS selected by a physician. Furthermore, it has been reported that the shape of the cornea is different between Asians and Caucasians.^23^ Therefore, it might be required that a separate CLS instruction manual for Asians.

## Conclusion

After the removal of the CLS, the subject of the present study noted transient blurred vision with aggravation of myopia and astigmatism. Furthermore, formation of central island-like pattern was shown by topographical analysis, due to orthokeratological effects by tight fitting of CLS. These findings resolved within 24 hrs after the removal of the CLS.
